# A Comparative Evaluation of Performance of Sysmex XN 3000 and Horiba Yumizen H2500 Automated Complete Blood Count Analysers

**DOI:** 10.1007/s12288-023-01687-6

**Published:** 2023-08-17

**Authors:** Rajesh Kumar Bhola, Christophe Fudaly, Shubham Rastogi

**Affiliations:** 1 Laboratory Haematology Division, Department of Pathology, IMS and SUM Hospital, S’O’A Deemed to Be University, Bhubaneswar, Odisha 751003 India; 2 Horiba Medical, HORIBA ABX SAS, Parc Euromédecine, Rue du Caducée, BP 7290, 34184 Montpellier Cedex 4, France

**Keywords:** Automated haematology analyser, Sysmex XN 3000, Yumizen H2500, Complete blood count

## Abstract

Modern automated laboratory haematology analysers use various methods to measure different haematological parameters. These parameters are useful in the diagnostic and clinical interpretation of patient symptoms. So, it is very important to compare the performance of different analysers measuring the same parameter. Hence, a comparison of complete blood counts analysed by Sysmex XN 3000 and Horiba Yumizen H2500 was performed. Total 296 EDTA anti-coagulated blood samples were processed in both the analysers in duplicate within 4 h of collection. The white blood cell count, red blood cell count, erythrocyte indices, differential leukocyte count, platelet count and platelet indices and reticulocyte count were compared. A good level of correlation and agreement between different parameters were obtained. A strong correlation was observed (r > 0.9) between Sysmex XN 3000 and Yumizen H2500 for WBC (0.997), RBC (0.997), Haemoglobin (0.999), haematocrit (0.974), MCV (0.902), MCH (0.99),, platelet count by impedance (0.989), mean platelet volume (0.954), plateletcrit (0.971), platelet distribution width (PDW) (0.916), neutrophils (0.997), lymphocytes (0.989), monocytes (0.943), and eosinophils (0.991) counts. A moderate correlation was observed for RDW-CV (0.75). The basophils count showed poor correlation (r < 0.5) possibly because of sample selection with mostly low basophils count. An acceptable bias was observed for most of the parameters like WBC, RBC, Haemoglobin, Haematocrit, platelet counts, neutrophils, lymphocytes, eosinophils and monocytes. The studied instruments ensured satisfactory interchangeability except for few parameters, thus facilitate substitution of one analyser by another without affecting the clinical decision making.

## Introduction

The Complete blood count (CBC) plays an important role in the routine haematological investigations of patients. The analysis of abnormalities within the white blood cells (WBC), red blood cells (RBC), and platelets (PLT) of peripheral blood is helpful in clinical interpretation of patients’ signs and symptoms [1]. Modern automated analysers allow the measurement of different haematological parameters in a more objective and precise manner compared to manual methods. With each passing day newer technologies are being introduced to produce more reliable and accurate results [2].

The manual microscopy is considered as the most reliable and the reference method for WBC evaluation and its differential counts when performed by experienced and expert microscopic morphologists. It is a time‐consuming and the interpretation of results depends on the number of cells included in the analysis as well as on the experience of the laboratory diagnostician [3]. As total number of leukocytes evaluated under the microscope varies from 100 to 500 cells, it has a high coefficient of variation (CV) including inter-personnel subjectivity too. Whereas the current generation automated analysers can perform counts on more than 1000 samples per day. It performs a differential count on nearly 8000–10,000 leukocytes which reduces the CV and removes subjective changes. Thus the results obtained are more reliable and reproducible [4, 5]. But different laboratories use different automated analysers from different in vitro diagnostic (IVD) manufacturers. And different IVD manufacturers use different technology to enumerate the same CBC parameter [6, 7] As all patients should receive same level of care irrespective of the analysers being used, it is required that the cell counters should produce comparable results and used interchangeably without affecting clinical decision making. Hence comparative analyses of different automated haematology analysers have previously been performed, and these have indicated variable differences in assaying either peripheral blood samples or body fluid specimens [2, 4, 6, 8]. Hence we compared the performance of two advanced haematology analysers: Sysmex XN‐3000 and Horiba Yumizen H2500.

## Materials and Methods

A prospective study has been carried out to compare the performance of different CBC parameters between Sysmex XN 3000 and Horiba Yumizen H2500 fully automated CBC analysers after obtaining institutional ethical clearance.

### Selection of Instrument

We have selected two instruments introduced into our laboratory for comparison, i.e., Sysmex XN 3000 and Horiba Yumizen 2500 which works on following technology.

The *Sysmex XN‐3000* (Sysmex, Kobe, Japan) uses different principle, methods and reagents to enumerate the CBC. The RBC and platelets are counted via Hydrodynamic focusing and impedance. The RBC is calculated as a particle count between two discriminators (lower discriminator, LD and upper discriminator, UD) which are set up in the ranges of 20–75 and 200–250 fl respectively. The RDW-SD is the distribution width at 20% frequency level with peak height assumed to be 100%. Similarly, RDW-CV is calculated from 68.26% of total distribution area. The haematocrit is calculated via the RBC pulse height detection method. SLS-haemoglobin method is used for haemoglobin estimation with use of Sulfolyser reagent which contains Sodium lauryl sulfate 1.7 g/L. It has advantage of being fast, non-poisonous and ability to analyse meth-haemoglobin too. The MCV, MCH and MCHC are calculated parameters. The platelet count is calculated as a particle count between two discriminators, LD and UD, set up in the ranges of 2–6 and 12–30 fl, respectively. The PDW is the distribution width at the 20% frequency level. The platelet large cell ratio (P-LCR) is the ratio of large platelet from the 12 fL discriminator or larger. MPV is a calculated parameter. Using flow cytometry method platelets can also be counted based on fluorescence i.e. PLT-F using Oxazine dye. The platelets showing strong fluorescent light intensity are reported as immature platelet fraction (IPF). Reticulocytes are also enumerated using flow cytometry method and polymethine dye. The WBC is analysed based on flow cytometry by hydrodynamic focusing where a semi-conductor laser beam (wavelength 633 nm) is emitted to the blood cells passing through the flow cell creating a forward scatter and side scatter and side fluorescent light captured by photodiode and avalanche photodiode respectively. The forward scatter provides information about size of blood cells, side scatter about internal complexity of cells like nuclear size and cytoplasmic characteristics and side fluorescence about degree of staining or activity. It uses different channels for enumeration of different population of cells, e.g., the WNR channel to count the WBC, nucleated RBC and basophils, WDF channel for classifying different WBC populations like lymphocytes, monocytes, neutrophils + basophils, and eosinophils. These channels use similar reagents consisting of organic quaternary ammonium salts, non-ionic surfactant polymethine dye, ethylene glycol but at different concentrations.

The *Horiba Yumizen H2500* (Horiba Medical, France) also uses different principle and technology to measure different CBC parameter. The RBC and platelets are counted using impedance method. The blood is diluted in the isotonic electrolyte i.e., ABX diluents. When a cell passes through a calibrated micro aperture, there is increase in electric resistance proportionate to the cell volume and representative histograms are generated. RBC histograms are presented by the distribution curves on 256 channels from 30 to 300 fL. Similarly platelet histogram is indicated by the distribution curve from 2 fL to a mobile threshold according to the microcyte population present in the analysis. The MCV and MPV are calculated as measured average from the respective distribution curves. For HGB measurement, ABX Lysebio reagent is used to lyse the erythrocytes, and stabilize the heme iron and measured spectrophotometrically at 555 nm wavelength. HCT, MCH, MCHC and Plateletcrit are calculated parameters. PDW, RDW-CV, RDW-SD, Microcytic cells % (MIC%), macrocytic cells ((MAC%) are calculated from RBC and Platelet histograms. The differential leukocyte count integrates multiple principles or techniques to determine the total nucleated cells (TNC) and separating NRBC from the WBC and different WBC subpopulation. The basophils are counted using Impedance method and ABX Basolyse reagents which lyse the membrane of all leucocytes except basophils. The lymphocytes, monocytes, neutrophils, eosinophils (LMNE) and NRBC are detected using Yumizen Nucediff reagent which lyse the RBC and stabilizes the WBC in its native forms and measured based on the principle of Double Hydrodynamic Sequential System (DHSS) flow cytometry. A matrix separates different population based on cell volume detected by impedance change and absorbance determined from optical transmission of light through the cell. The WBC are counted in three different chambers: TNC/HGB channel, BASO/TNC2 channel and LMNE channel. Reticulocytes are counted using flow cytometry technology which combines two methodology of Impedance for volume and orthogonal fluorescence signal. It uses ABX Fluocyte reagent which contains fluorescent stain—Thiazole orange which is specific to nucleic acid, i.e., RNA in reticulocytes. Optical platelets detection method also uses ABX Fluocyte reagent and based on flow cytometry technology using Impedance and absorbance methodology.

Both the instruments were installed in the laboratory haematology section of the institution with performance verification done as per the standard protocol. The Sysmex XN 3000 analyser was calibrated using XN CAL. The Yumizen H2500 instrument was calibrated using ABX Minocal. Daily quality control (QC) were run in the laboratory with XN Check Level L1, L2, and L3 for Sysmex XN 3000 and DIFFTROL L, N and H (Low, normal and High) for Yumizen H2500.

### Sample Size Calculation [9]

The sample size calculation for agreement between two methods of measurement was calculated using the following formula where standardized difference limits ($\mu /\sigma )$ = 0.1, standardized agreement limits ($\delta /\sigma$) = 2.5, type II error (*β*) = 0.2 and *α* = 0.05.

$$n=\frac{\left(2+{z}_{1-\gamma /2}^{2}\right){\left[tinv(1-\beta /2,n-1,{t}_{1-\alpha /2,n-1})\right]}^{2}{S}_{D}^{2}}{2{\left({z}_{1-\gamma /2}{S}_{D}-\delta \right)}^{2}}$$

We got the sample size to be 271 and considering 10% to adjust for any outlier exclusion, the total sample size determined to be 298 (rounded off 300). During data analysis, 4 samples were excluded from the data for missing data points and outliers. So total 296 samples were selected and included in the study.

### Sample Collection and Processing

A total of 296 K_2_EDTA anticoagulated peripheral blood samples were collected by evacuated tube system (ETS) irrespective of age and gender. 10–20 samples were selected randomly from the daily sample pool and anonymized and deidentified. All the samples were analysed in duplicate in Sysmex XN 3000 in CBC + Diff + Retic + PlatF mode and Horiba Yumizen H2500 in CBC + Diff + Retic mode. All the samples were processed within 4 h of sample collection.

### Blood Smear Analysis

Peripheral blood smears were prepared automatically and stained on Sysmex SP10 automated slide maker and Stainer to avoid a human error in the context of discrepancies in slide interpretation due to different smear technique. Each blood sample and slide were coded with a unique numerical identifier which provides patients with anonymity and simultaneously allows the comparison of examination results. Differentiation of white blood cells on smears was performed by specialized diagnostician on 200 cells (leukopenia samples—WBC < 2 × 10^9^/l—were analyzed up to 100 cells if possible) as per the CLSI guidelines [10].

### Statistical Analysis

Statistical analysis was performed with Microsoft Excel (Microsoft Corporation, Redmond, WA). A degree of agreement between the same parameters analysed with two haematology analysers was evaluated using the non‐parametric Passing and Bablok regression method. The regression equation is presented as y = ax + b where the proportional difference between two methods is represented by slope (b) and the constant difference is represented as the regression line’s intercept, the constant ‘a’. The confidence interval (95% CI) explains if the value of constant differs from zero and the slope differs from 1 only by chance or not. If the CI includes 0 for the constant it can be concluded that there is no constant bias between the methods. Similarly, if the CI includes 1 then it can be concluded that there is no proportional bias between the two methods. That means y = x so that both methods can be used interchangeably. Otherwise, corrections need to be applied in those cases where the values 1 for slope and 0 for intercept not enclosed in the respective confidence interval indicative of significant deviation. The method comparison is also done by Bland and Altman analysis. A *P* value < 0.05 was considered to be statistically significant for every analysis. For all statistical analysis Sysmex XN analysers value was considered as reference method for comparison.

## Results

Total 296 peripheral blood samples were analysed on both Sysmex XN 3000 and Horiba Yumizen H2500 and the results were compared as depicted in Table 1. The distribution of different parameters is spread across different ranges of values.

**Table 1:** Comparison of different parameters on Sysmex XN and Horiba H2500 depicting the minimum value, the maximum value, the average, and standard deviation of all samples processed by both the analyzers

Parameter	XN Min	YH Min	XN Max	YH max	XN average	YH average	XN SD	YH SD
WBC	0.27	0.24	34.45	33.57	9.49	9.25	3.95	3.84
RBC	1.75	1.68	7.68	7.56	4.60	4.57	0.87	0.85
HGB	4.85	4.86	17.75	17.76	12.31	12.35	2.27	2.27
HCT	13.25	14.62	52.40	54.26	37.58	38.09	6.56	6.83
MCV	61.20	60.50	102.35	102.75	82.29	83.89	7.28	7.72
MCH	17.85	17.75	32.95	33.74	26.97	27.22	2.91	2.92
MCHC	28.05	28.54	36.60	34.09	32.73	32.40	1.32	0.70
RDW CV	11.85	11.58	23.95	21.74	14.67	14.68	2.09	1.80
PLT	6.00	12.57	709.00	650.58	263.29	253.53	97.04	92.87
MPV	8.60	7.58	15.20	14.41	12.01	11.27	1.36	1.30
PCT	0.05	0.04	0.79	0.71	0.32	0.29	0.10	0.09
PDW	8.75	10.50	25.10	35.44	15.98	19.63	3.64	4.69
Neutrophils (#)	0.14	0.07	32.16	30.00	6.40	6.22	3.76	3.61
Lymphocytes (#)	0.10	0.11	6.06	5.43	2.22	2.16	0.93	0.88
Monocytes (#)	0.03	0.02	1.60	1.56	0.42	0.42	0.18	0.18
Eosinophils (#)	0.00	0.01	3.08	2.99	0.39	0.38	0.47	0.44
Basophils (#)	0.00	0.00	0.13	0.28	0.03	0.03	0.02	0.03

*XN* Sysmex XN 3000, *YH* Horiba Yumizen H2500, *min* minimum, *max* maximum, *SD* standard deviation

**Fig. 1:**
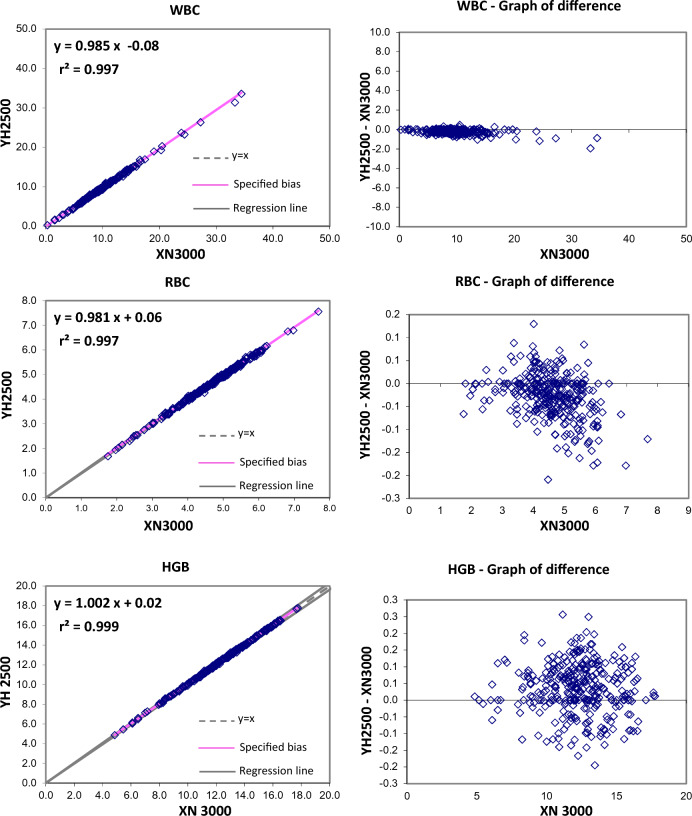

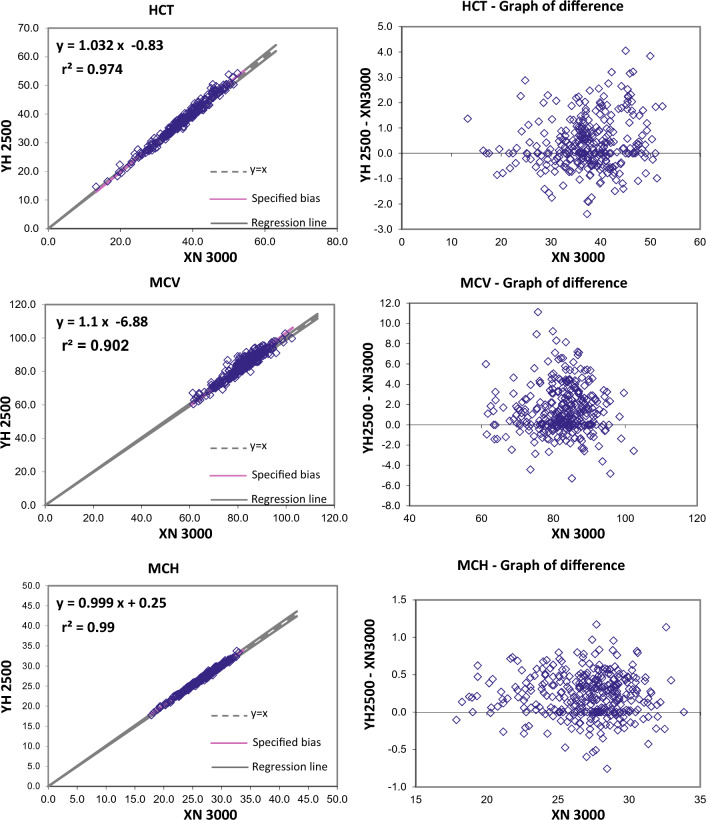

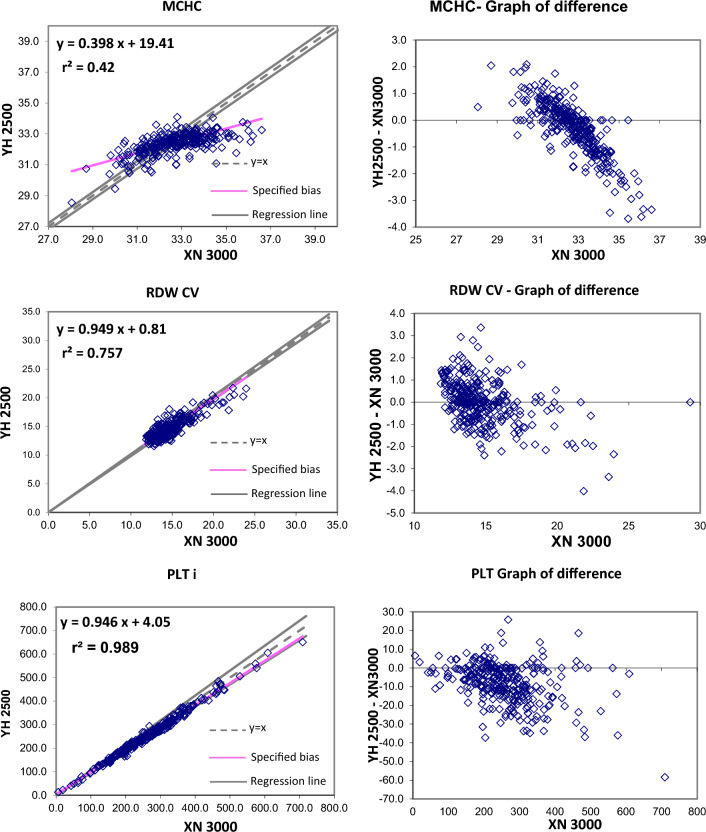

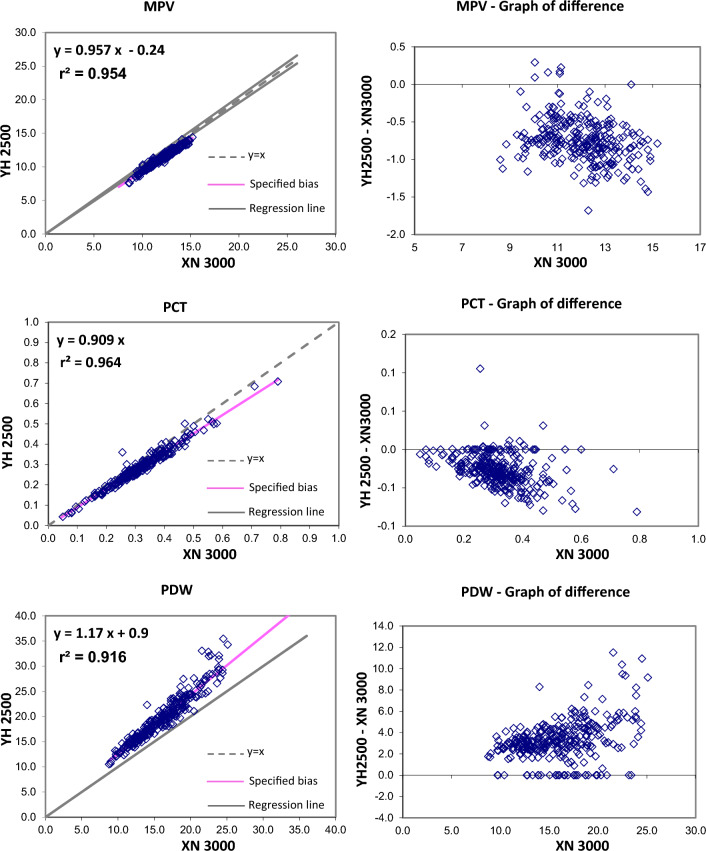
Passing and Bablok regression analyses of different CBC parameters – WBC, RBC, HBG, HCT, MCV, MCH, MCHC, RDW-CV, PLT, MPV, PCT, PDW analyzed in YH 2500 and XN 3000

**Fig. 2:**
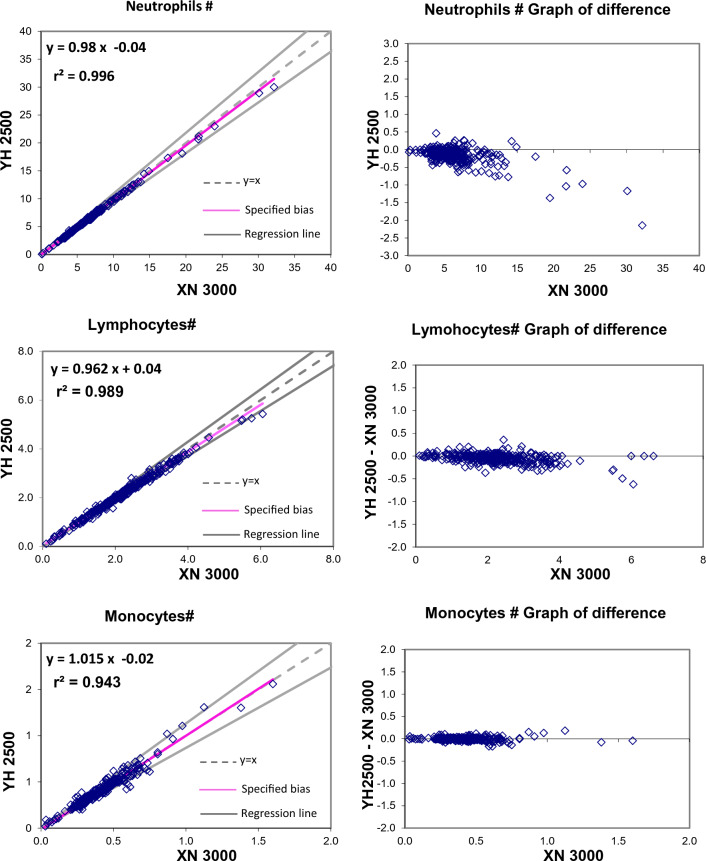

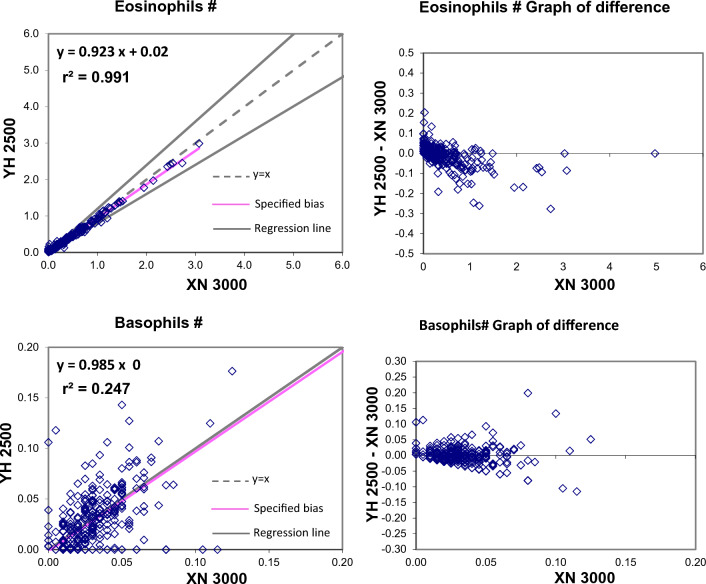
Passing and Bablok regression analyses of WBC differential counts—Neutrophils%, Lymphocytes, Monocytes, eosinophils%, basophils % and NRBC analysed in YH 2500 and XN 3000

The Passing and Bablok regression analysis for agreement between the two different analysers showed good agreement for most studied parameters. Table 2 present exact equations and 95% confidence intervals for slope and intercept for all arrangements. The corresponding scatter plots are presented in the Fig. 1. When the parameters were compared between YH2500 and XN3000, there were strong positive correlation for WBC, RBC, HGB, HCT MCV, MCH, platelet count, MPV, PCT, PDW, neutrophils, lymphocytes, monocytes, eosinophils. There was good correlation between RDW-CV whereas MCHC showed moderate correlation and basophils count showing weak correlation. The Passing and Bablok regression analysis showed that there was good agreement between most of the parameter except MCHC and basophils. MCHC showed a high constant bias with low proportional bias. Similarly, basophils showed a small constant bias with a proportional bias. The scattergram with regression line and confidence bands for regression line and the residual plots with distribution of difference around the fitted regression line have been depicted in the Figs. 1 and 2.

**Table 2:** Passing Bablock regression analysis of different CBC parameters

Parameter	Number (N)	Regression coefficient (r^2^)	Equation for y = ax + b	SE	95% CI for intercept (b)	95% CI for slope (a)
WBC	295	0.997	0.985x − 0.08	0.2096	− 0.155 to 0.041	0.970 to 0.993
RBC	296	0.997	0.981x + 0.06	0.0465	0.025 to 0.104	0.971 to 0.989
HGB	296	0.9988	1.002x + 0.02	0.0786	− 0.014 to 0.133	0.993 to 1.005
HCT	296	0.9743	1.032x − 0.83	1.0967	− 2.228 to − 0.056	1.011–1.073
MCV	296	0.902	1.1x − 6.88	2.4261	− 9.666 to − 1.017	1.028 to 1.135
MCH	296	0.99	0.999x + 0.25	0.2964	− 0.255 to 0.682	0.984 to 1.019
MCHC	296	0.420	0.398x + 19.41	0.5360	15.970–20.940	0.352 to 0.504
RDW-CV	295	0.757	0.949x + 0.81	0.8892	− 0.008 to 2.943	0.800 to 1.005
PLT-I	293	0.9885	0.946x + 4.05	9.9677	− 2.051 to 7.535	0.931 to 0.971
MPV	276	0.954	0.957x − 0.24	0.2801	− 0.654 to 0.150	0.924 to 0.991
PCT	276	0.964	0.909x	0.0171	− 0.011 to 0.006	0.88 to 0.94
PDW	276	0.9157	1.17x + 0.9	1.3645	− 0.787 to 0.915	1.162 to 1.276
Neutrophils (#)	295	0.9961	0.98x − 0.04	0.2263	− 0.078 to 0.047	0.964 to 0.989
Lymphocytes (#)	291	0.9891	0.962x + 0.04	0.0921	0.007 to 0.082	0.938 to 0.974
Monocytes (#)	285	0.9430	1.015x − 0.02	0.0439	− 0.021 to 0.010	0.946 to 1.033
Eosinophils (#)	294	0.9908	0.923x + 0.02	0.0421	0.015 to 0.025	0.899 to 0.939
Basophils (#)	296	0.2313	2.161x − 0.43	0.3393	− 0.520 to − 0.263	1.693 to 2.455

The shaded cells indicate either the 95% CI for slope doesn’t include 1 or intercept doesn’t include 0 statistically

The degree of difference obtained by Bland and Altman analysis for different parameters shows acceptable bias or within systematic error limits for WBC, RBC, HGB, HCT, MCH, RDW CV%, Platelets, lymphocytes %, eosinophils %. MCV shows a marginal higher value by Yumizen H2500 in comparison to XN 3000. MCHC shows a higher value by YH2500 at a lower value whereas a negative bias observer at higher MCHC. MPV shows a negative bias. When the differential leukocyte count is compared, neutrophils showed a negative bias. Basophils and NRBCs showed a positive systematic bias (Table 3).

**Table 3:** Bland and Altman analysis

Parameter with bias specification		Level 1								Level 2							Level 3							
		Ref test		Test		Bias		CI		Ref test		Test		Bias		CI	Ref test		Test		Bias		CI	
WBC (5.60%)		2.00		1.89		− 5.58%		− 8.5 to − 1.0%		8.00		7.80		− 2.53%		− 2.8 to − 2.3%	30.00		29.46		− 1.79%		− 2.9 to − 1.2%	
RBC (1.70%)		2.00		2.02		1.07%		0.1 to 2.3%		4.00		3.98		− 0.43%		− 0.5 to − 0.2%	6.00		5.94		− 0.92%		− 1.2% to 0.7%	
HBG (1.80%)		6.00		6.03		0.56%		0.3 to 1.5%		12.00		12.05		0.41%		0.3% to 0.5%	17.00		17.06		0.36%		0.1% to 0.4%	
HCT (1.70%)		30.00		30.13		0.45%		− 0.3% to 1.1%		40.00		40.46		1.14%		0.9 to 1.9%	55.00		55.94		1.71%		1.1 to 3.3%	
MCV (1.20%)		70.00		70.15		0.22%		− 0.6 to 1.5%		90.00		92.16		2.40%		1.6 to 2.7%	110.00		114.17		3.79%		1.9 to 4.6%	
MCH (1.40%)		25.00		25.23		0.91%		0.8 to 1.3%		30.00		30.22		0.74%		0.7 to 1.1%	35.00		35.22		0.62%		0.4 to 1.2%	
MCHC (0.80%)		28.00		30.56		9.14%		7.4 to 10.0%		33.00		32.55		− 1.36%		− 1.4 to − 1.1%	36.00		33.75		− 6.26%		− 6.6 to − 5.3%	
RDW CV%		10.00		10.86		8.58%		0.3 to 9.3%		12.00		12.51		4.26%		0.3 to 4.5%	16.00		15.82		− 1.13%		− 1.7 to 0.6%	
RDW CV% (1.70%)		10.00		10.30		3.04%		0.4 to 9.5%		12.00		12.20		1.69%		0.4 to 4.6%	16.00		16.00		− 0.01%		− 1.8 to 0.5%	
Platelets (5.90%)		50.00		51.35		2.69%		− 7.1 to 8.2%		200.00		193.24		− 3.38%		− 4.2 to − 2.9%	600.00		571.62		− 4.73%		− 5.7 to − 3.2%	
MPV (2.30%)		6.00		5.50		− 8.37%		− 11.7 to − 5.2%		9.00		8.37		− 7.02%		− 8.2 to − 6.0%	12.00		11.24		− 6.34%		− 6.6 to − 6.2%	
PDW		10.00		12.61		26.05%		19.5 to 25.6%		15.00		18.46		23.04%		21.7 to 23.2%	20.00		24.31		21.53%		20.8 to 23.8%	
Neutrophils # (9.10%)		2.0		1.92		− 3.81%		− 5.0 to − 1.2%		5.0		4.86		− 2.73%		− 3.0 to − 2.4%	9.0		8.78		− 2.41%		− 3.1 to − 2.0%	
Lymphocytes # (7.40%)		1.00		1.00		− 0.18%		− 2.32 to 2.0%		2.50		2.44		− 2.32%		− 3.1 to − 2.2%	3.50		3.40		− 2.73%		− 3.9 to − 2.4%	
Monocytes # (13.20%)		0.20		0.19		− 6.45%		− 7.4 to − 0.4%		0.50		0.49		− 1.68%		− 3.6 to − 0.8%	1.0		1.0		− 0.09%		− 4.4 to 1.2%	
Eosinophils # (19.80%)		0.15		0.16		6.87%		2.9 to 6.87%		0.50		0.48		− 3.35%		− 5.6 to − 2.7%	1.20		1.13		− 5.90%		− 8.2 to − 4.7%	
Basophils # (15.40%)		0.20		0.22		10.55%		− 8.4 to 37.9%		0.50		0.56		11.94%		− 8.1 to 42.5%	–		–		–		–	

## Discussion

It is encouraging to find that a satisfactory agreement was seen between most of the analysed parameters, but some of the parameters show constant and /or systematic bias indicating that Sysmex XN 3000 and Horiba Yumizen H2500 are not fully interchangeable in terms of CBC analysis. Except for HGB, MCH and RDW-CV, none of the parameter showed by regression analysis to have 95% confidence interval of intercept and slope of 0 and 1 respectively within it. But when the bias was calculated for 3 different types of values (Low, medium and high values), MCV, MCH, MCHC, RDW CV, MPV and basophils have reported higher bias than the acceptable limits. For WBC count and neutrophil count a higher negative bias is reported by YH2500 in comparison to XN 3000 analyser.

When the basic cell counts like RBC, HGB, Hct, WBC and Platelets were compared, there are strong positive correlation and acceptable bias with negligible systematic error which indicates that these parameters can be used interchangeably within the laboratory. *Milena Malecka et. al.*, have also reported consistent results between for these parameters [11]. Similarly good agreement was observed between Sysmex XN 1000 and Mindray BC-5180; Mindray BC-6800, Sysmex XN 2000, and Beckman Coulter LH750; Sysmex XN 3100 and Sysmex XE 2100 [12–14]. But Mathias Bruegel et.al. found systematic difference for WBC count among the analysers with a relatively higher count by Advia2120i. The platelet count measured by Cell-Dyn Sapphire and Advia2120i were higher whereas lower for DxH800 and XE-5000 [8].

When the RBC and platelet indices are compared, good agreement was observed for MCV, MCH, RDW-CV%, MPV, PCT and PDW except MCHC. There was a high constant bias with positive proportional bias observed in MCV. The percentage bias was high for high MCV values. The bias estimated at low MCHC was 9.14%, normal MCHC -1.36% and high MCHC 6.26%. Incompatibility for MCHC between XN and Yumizen H2500 has been reported by Malecka et al. too [11]. But additionally, they have found incompatibility for PDW compared to our study which showed good agreement. Hence a caution is recommended while interpreting MCHC and PDW values reported by different analysers for clinical management purpose as they can’t be used interchangeably. In many centres including our centre doesn’t report PDW and still the widespread clinical usefulness of it needs to be established.

The comparison of WBC differential leukocyte count between XN and Yumizen H2500 showed a good correlation except for Basophils count. But the regression analysis indicates that there was statistically significant constant bias and proportional bias between XN and Yumizen H2500. The Yumizen H2500 showed a negative constant bias with proportional bias for neutrophils count in comparison for XN. Lymphocyte counts showed minor constant bias without proportional bias as 95% of CI of slope includes 1. Whereas monocyte counts showed a good agreement. Similarly, eosinophils showed a positive constant and proportional bias. Basophils showed a negative constant and high proportional bias. Neutrophils, lymphocytes, monocytes and eosinophils have acceptable bias specifications. But basophils showed higher bias by Yumizen H2500 possibly because most of the samples had a very low basophil count. In contrast, *Milena Malecka et. al.* has found eosinophils showing more variation by Yumizen H2500 in comparison to XN. Other differential counts showed good agreement [11]. Lisa Meintker et al. compared the differential leukocyte count between Abbott Sapphire, Siemens Advia 120, Sysmex XE-2100, and Beckman Coulter DxH 800 and found very good correlation for neutrophils and eosinophils, fair correlation for lymphocytes and monocytes and very poor correlation for basophils count. The lymphocyte count showed minimal systematic bias between Sapphire, XE-2100 and DxH 800 whereas Advia 120 reported lower lymphocyte count. Similarly Advia 120 reported lower monocyte count and DxH reported higher monocyte count in comparison to average count [15]. Similarly the results obtained by Sysmex XN3100 correlated well with Sysmex XE-2100 except monocyte count and basophil count which showed mean difference of 16.5 and 38 respectively [14].

In conclusion, the results obtained from different automated CBC analyzers in questions are similar. For few of them reference ranges and cut off values will need to be redefined according to instrument technology as we have observed slight constant and/or proportional bias for different parameters though mostly within acceptable limits.
